# Calcium Propionate Supplementation Has Minor Effects on Major Ruminal Bacterial Community Composition of Early Lactation Dairy Cows

**DOI:** 10.3389/fmicb.2022.847488

**Published:** 2022-03-08

**Authors:** Fan Zhang, Yue Wang, Hui Wang, Xuemei Nan, Yuming Guo, Benhai Xiong

**Affiliations:** ^1^State Key Laboratory of Animal Nutrition, Institute of Animal Science, Chinese Academy of Agricultural Sciences, Beijing, China; ^2^State Key Laboratory of Animal Nutrition, College of Animal Science and Technology, China Agricultural University, Beijing, China

**Keywords:** calcium propionate, dairy cows, early lactation, rumen fermentation, negative energy balance, ruminal bacterial community composition

## Abstract

Calcium propionate is one kind of good source for preventing and treating hypocalcemia and ketosis for dairy cows in early lactation. However, little is known about the effects of different feeding levels of calcium propionate on the ruminal bacterial community of early lactation dairy cows. This study aimed to explore the effects of different calcium propionate feeding levels on the ruminal fermentation and bacterial community composition of early lactation dairy cows. Twenty-four multiparous cows were randomly allocated into control (CON), low calcium propionate (LCaP), medium calcium propionate (MCaP), and high calcium propionate (HCaP) groups with six cows per group after calving. The CON group cows were fed the normal total mixed ration (TMR), and the cows of the LCaP, MCaP, and HCaP groups were fed TMR supplemented with 200, 350, and 500 g/day calcium propionate for 35 days after calving, respectively. The rumen fermentation parameters were measured every week, and the ruminal bacterial community composition of the last week was analyzed by 16S rRNA gene sequencing. Under the same diet, the rumen pH showed no difference among the four groups, but the content of microbial crude protein (MCP) and ammonia nitrogen quadratically decreased and linearly increased with calcium propionate supplementation, respectively. The feeding of calcium propionate linearly increased the concentrations of total volatile fatty acid (VFA), acetate, propionate, butyrate, iso-valerate, and valerate in the rumen. In all the treatment groups, *Bacteroidetes*, *Firmicutes*, and *Proteobacteria* were the dominant phyla, and *Prevotella_1* and *Succiniclasticum* were the dominant genera in the rumen. Compared with the CON group, the addition of calcium propionate to the early lactation dairy cows quadratically improved the alpha diversity index of Chao1 estimator and observed species, but had little effect on the relative abundance of the major bacterial at phyla and genera level. These results suggested different levels of calcium propionate supplementation improved the rumen fermentation and the ruminal bacterial diversity but had little impact on the major ruminal bacterial community composition of dairy cows in early lactation.

## Introduction

The most important challenge for early lactation dairy cows is the difficulty of nutrient intake to meet the nutritional requirements of rapidly increasing milk production. Dairy cows in this period presented complicated metabolic challenges with blood calcium defects and negative energy balance (NEB), especially high-performing dairy cows. Hypocalcemia is a known risk for many diseases, including milk fever, dystocia, uterine prolapse, retained placenta, metritis, displaced abomasum, ketosis, and mastitis (Couto Serrenho et al., 2021). Cows with severe NEB are susceptible to fatty liver, ketosis, and other metabolic disorders. Therefore, hypocalcemia and NEB play etiological roles in the reduction in dry matter (DM) intake and milk production, reproductive disorders development, fertility problems, and infectious diseases (Kara, 2013), which should be prevented and treated immediately for the periparturient cows. Increasing dietary calcium and energy feeding levels is a common method to prevent and treat hypocalcemia and ketosis for early lactation dairy cows. In ruminants, propionate serves as an energy source that contributes to 60–74% of hepatic gluconeogenesis (Aschenbach et al., 2010). Calcium propionate, which can be hydrolyzed into propionic acid and calcium ions in the rumen, is widely used to correct the metabolic problem in dairy cows (Zhang et al., 2017). Goff et al. (1996) observed dairy cows administered with one calcium propionate tube (each containing 37 g of calcium) respectively at calving and again 12 h after calving was beneficial in reducing subclinical hypocalcemia and the incidence of milk fever. Liu et al. (2010) reported that different amounts of calcium propionate (100, 200, and 300 g/day) supplementation improved the nutrient digestibility and energy status of dairy cows in early lactation. In the study conducted by Martins et al. (2019), Holstein cows fed calcium propionate (200 g/day) increased milk yield during early lactation. In addition, we reported that dietary supplementation with calcium propionate to the dairy cows in early lactation quadratically increased milk yield and DM intake, and the optimum amount of calcium propionate was 350 g/day (Zhang et al., 2022). We speculated that the benefit of improved lactation performance after feeding calcium propionate might be related to the process of rumen microbial fermentation.

In ruminants, a wide range of microorganisms carry out extensive and complex metabolic activities, fermenting plant proteins and polysaccharides to produce nutrients for ruminant maintenance, growth, and lactation (Liu et al., 2019). The rumen microbiome directly or indirectly contributes to animal production and is susceptible to changes in feed types and components of the diet (Kataev et al., 2020). The profile of the rumen microbial community and the abundance of metabolites (including the rumen fermentation index) play key roles in the health status and production performance of dairy cows (Wang et al., 2021b). A better understanding of the changes of rumen microbial community compositions is crucial to invest the impact of calcium propionate feed additive in dairy cows. Many studies about the application of calcium propionate in dairy cows focused on lactation performance and metabolic properties, whereas the effect of calcium propionate on rumen fermentation and ruminal bacterial community composition had received less attention (Goff and Horst, 1993; Pehrson et al., 1998; Liu et al., 2010). As one of the major fermentation productions of rumen microbiota from carbohydrates, the supplementation of propionate-related production might affect the rumen fermentation profiles and ruminal bacterial communities. The 16S rRNA sequencing is a well-tested, fast and cost-effective method for analyzing the differential abundance of microbial communities (Liu et al., 2019). Therefore, this study aimed to evaluate the application of calcium propionate as a calcium source and glycogenic precursor during early lactation on rumen fermentation and ruminal bacterial community composition in high-yielding dairy cows.

## Materials and Methods

The animals used in the study were approved by the Animal Care Committee of the Chinese Academy of Agricultural Sciences (Beijing, China; No. IAS2020-93), and all experimental procedures were following the guidelines of the academy for animal research.

### Animals and Diets

This study was performed at the China-Israel demonstration dairy farm (Beijing, China) from September to December 2020. Twenty-four multiparous Holstein cows in early lactation were randomly divided into four blocks (*n* = 6 per treatment) based on parity (2, *n* = 4; 3, *n* = 12; and 4, *n* = 8), previous lactation corrected 305-days milk yield (12,672 ± 312 kg), and anticipated calving date. All the dairy cows were fed the same basal total mixed ration diet (TMR), which was formulated according to the National Research Council (NRC, 2001) recommendation. The ingredients and chemical composition of the diet are shown in Table 1. The four treatments of control (CON), low calcium propionate (LCaP), medium calcium propionate (MCaP), and high calcium propionate (HCaP) groups were oral drenched with 0, 200, 350, and 500 g/day calcium propionate (Jiangsu Runpu Food Technology Co. LTD., Lianyungang, Jiangsu, China) per cow from calving to days 35 postpartum, respectively. The calcium propionate was oral drenched three times a day in equal amounts after milking. During the experiment, the cows were milked three times a day at 6:00, 14:00, and 22:00, and the TMR was fed *ad libitum* after milking. The TMR was adjusted according to the orts and made sure there were 5% ~ 10% orts every day. The cows were housed in individual stalls and had free access to water.

**Table 1:** Composition and nutrient levels of the experiment diet [dry matter (DM) basis].

Ingredients	Composition (%)	Nutrient level	Composition
Sprouting corn bran	2.19	DM (%)	50
Stem-flaked corn	3.58	CP (%)	17.70
Cotton seed	2.28	NE_L_^3^, Mcal/kg	1.72
Megalac^1^	0.50	NDF (%)	28.01
Fat powder	1.14	ADF (%)	15.87
Pelleted beet pulp	1.31	EE (%)	4.17
Wet brewers’ grains	3.73	Ash (%)	9.07
Alfalfa	9.90	Ca (%)	0.85
Oat hay	2.16	P (%)	0.42
Concentrate^2^	41.93		
Corn silage	31.31		

1 The megalac is a rumen-protected fat supplement. (Volac Wilmar Feed Ingredients Ltd, Hertfordshire, United Kingdom).

2 The concentrate for postpartum dairy cows was manufactured by Beijing Sanyuan Seed technology Co., Ltd (Beijing, China). The nutrient levels of the concentrate: DM, 88.50%; CP, 23.91%; NDF, 13.20%; ADF, 7.40%; Ash, 13.1%; Ca, 1.41%; P, 0.58%; K, 1.20%; Mg, 0.58%; Na, 0.99%; Cu, 46.25 mg/kg; Fe, 80.30 mg/kg; Zn, 136.76 mg/kg; VA, 20.53 kIU/kg; VD, 3548.5 IU/kg; and VE, 116.9 IU/kg.

3 The NE_L_ was calculated according to NRC (2001).

The TMR samples were collected from the trough weekly on 2 consecutive days, composited to get one sample per week. The obtained samples were dried at 55°C for 48 h in an oven, and ground through a 1-mm screen for chemical composition analysis. The composition and nutrient levels of TMR were analyzed according to the methods of the Association of Official Analytical Chemists (AOAC, 2005). The DM was measured by drying the samples at 105°C for 3 h (method 934.01; AOAC, 2005). The nitrogen (N) content was determined by the Kjeldahl method and crude protein (CP) was calculated as 6.25 × N (method 954.01; AOAC, 2005). Ether extract (EE) was measured by the weight loss of DM after extraction with diethyl ether in a Soxhlet extraction apparatus for 8 h (method 920.39; AOAC, 2005). The content of ash was measured in a muffle furnace for 6 h at 550°C (method 942.05; AOAC, 2005). The calcium (Ca) was measured by an M9W-700 atomic absorption spectrophotometer (Perkin-Elmer Corp, Norwalk, CT, United States; method 968.08; AOAC, 2005). Total phosphorus (P) was analyzed by the molybdovanadate colorimetric method (method 946.06; AOAC, 2005). The neutral detergent fiber (NDF) was analyzed with heat-stable α-amylase and expressed inclusive of residual ash (Van Soest et al., 1991). The acid detergent fiber (ADF) content in TMR was determined according to the method 973.18 of AOAC (2005).

### Ruminal Sample Collection and Chemical Analyses

Ruminal fluid was collected from six cows of each group at days 7, 14, 21, 28, and 35 of parturition. Cows were sampled by an oral stomach tube and 200 ml syringe before morning feeding. The initial 150 ml was discarded to minimize saliva contamination. The ruminal pH value was measured immediately using a pH meter (Model 144 PB-10, Sartorius Co., Germany). The rumen fluid was filtered through four layers. The filtered liquid sample of about 10 ml was collected in a 15 ml centrifuge tube, acidified with 25% (w/v) metaphosphoric acid (5:1), and stored at −20°C for analysis of ruminal fermentation parameters. The concentrations of volatile fatty acids (VFA) profile were measured by the Agilent 6890 N gas chromatograph (Agilent Technologies Inc., Santa Clara, CA, United States) fitted with a capillary column (HP-FFAP19095F-123, 30 m × 0.53 mm diameter and 1 mm thickness, Agilent Technologies, Inc., Santa Clara, CA, United States; Jia et al., 2018). The ammonia nitrogen (NH_3_-N) was measured by the colorimetric method described by Lv et al. (2019) and microbial crude protein (MCP) was analyzed according to the method of Makkar et al. (1982). Then, 2 ml of the rumen fluid for each cow in the last time (days 35 postpartum) was collected in one sterile Eppendorf tube and stored in liquid nitrogen for DNA extraction.

### DNA Extraction, Amplification, and 16S rRNA Gene Sequencing

The microbial DNA of the ruminal fluid samples (days 35 postpartum) was extracted using a commercial DNA Isolation Kit (MoBio Laboratories, Carlsbad, CA, United States) according to the manufacturer’s instructions. The quality and purity of the genomic DNA were checked on 1% agarose gels and a NanoDrop 2000 UV spectrophotometer (ThermoFisher, Waltham, MA, United States). The V3-V4 hypervariable region of the bacterial 16S rRNA gene was amplified with the primers 338F (5′-ACTCCTACGGGAGGCAGCAG-3′) and 806R (5′-GGACTACNNGGGTATCTAAT-3′; Cui et al., 2019). For each ruminal sample, an eight-digit barcode sequence was added to the 5′ end of the forward and reverse primers (Allwegene Company, Beijing, China). The PCR was performed in a 25 μl reaction volumes, containing 12.5 μl 2 × Taq PCR Master Mix, 3 μl BSA (2 ng/μl), 1 μl Forward Primer (5 μM), 1 μl Reverse Primer (5 μM), 2 μl template DNA, and 5.5 μl dd H_2_O. The PCR cycling conditions were used as follows: 95°C for 5 min, followed by 28 cycles of 95°C for 45 s, 55°C for 50 s, and 72°C for 45 s with a final extension at 72°C for 10 min. The PCR amplicons products were extracted from 2% agarose gels and purified using an Agencourt AMPure XP Kit (Beckman Coulter, Brea, CA, United States). The purified amplicons were pooled in equimolar and paired-end sequenced (2 × 300) on an Illumina MiSeq platform (Illumina, San Diego, CA, United States).

### Statistical and Bioinformatics Analysis

The raw sequences data were first screened and the sequences were discarded if they were shorter than 120 bp, had a quality score (≤20) over a 50 bp sliding window, any contained ambiguous bases, or did not exactly match to primer sequences and barcode tags. Only sequences that overlapped >10 bp and had <10% mismatches were assembled (Bi et al., 2018). The assembled sequences were clustered into operational taxonomic units (OTUs) at a similarity level of 97% (Edgar, 2013). After splicing, we use VSEARCH (v2.7.1) software to remove tags with a length of less than 230 bp. The Ribosomal Database Project (RDP) Classifier tool was used to classify all sequences into different taxonomic groups against the SILVA128 database (Cole et al., 2009). Alpha diversity values including observed species, Chao1, PD_whole_tree, Shannon index, and Simpson index of the ruminal bacterial communities were calculated using the QIIME software (v1.8.0) based on the OTU information. Based on the results of taxonomic annotation and relative abundance, R (v3.6.0) software was used for bar-plot diagram analysis. The principal coordinate analysis (PCoA) and nonmetric multidimensional scaling analysis (NMDS) were performed based on Bray-Curtis distance by QIIME1 (V1.8.0) and R packages vegan (v3.6.0) software (Ran et al., 2021). The raw sequencing data of this study were available in the NCBI SRA database with the BioProject ID of PRJNA791506.^1^

### Statistical Analysis

Data about rumen fermentation parameters, the alpha diversity indices, the relative abundance of phylum and genus were analyzed using the MIXED procedure in SAS 9.4 (SAS Institute Inc., Cary, NC, United States). The repeated measures about rumen fermentation parameters included the fixed effects of treatment, lactation week, the interaction between diet treatment and lactation week, and the random effect of the block. The model about ruminal microbial data was analyzed with treatment as a fixed effect, and block as a random effect. The compound symmetry was used as the variance–covariance error structure in the process of analysis. Duncan’s multiple range tests were used to examine the significance among treatments. Orthogonal polynomial contrasts were used to determine the linear and quadratic responses to the increasing calcium propionate supplementation levels. The IML procedure of SAS was used to generate coefficients adjusted for the unequal spacing of calcium propionate supplementation in the diet. The data were presented as the least square mean and SEM. Statistical significance was assumed at *p* ≤ 0.05 and 0.05 < *p* ≤ 0.10 was designated as a tendency for all analyses.

## Results

### Rumen Fermentation Parameters of Different Dietary Treatments

The effects of calcium propionate supplementation on rumen fermentation of the 5 weeks were shown in Table 2. The calcium propionate supplementation did not affect the rumen fermentation of rumen pH, iso-butyrate, and the ratio of acetate to propionate. The concentration of MCP decreased quadratically (*p* = 0.02) with the increasing supplementation of calcium propionate. With the increasing feeding of calcium propionate, the content of NH_3_-N (*p* = 0.01) and total VFA (*p* = 0.05) increased linearly. Dietary supplementation with calcium propionate linearly increased the concentration of rumen acetate (*p* = 0.05), iso-valerate (*p* = 0.03), and valerate (*p* = 0.007), with the highest values in the HCaP group. Furthermore, a linear trend increase appeared in the concentration of propionate (*p* = 0.07) and butyrate (*p* = 0.07) as the calcium propionate dose increased. The molar proportions of individual rumen fluid VFA were shown in Supplementary Table S1. The molar proportions of acetate, propionate, and butyrate were not changed by the calcium propionate addition. However, the valerate proportion increased linearly (*p* = 0.004) with the dietary addition of calcium propionate.

**Table 2:** Effects of dietary supplementation with calcium propionate on ruminal pH and fermentation parameters of dairy cows in early lactation.

		Treatments^1^						*p*-value				
Items					SEM^2^
	CON	LCaP	MCaP	HCaP		Treatment	Linear	Quadratic	Week	Treatment × Week
pH	6.56	6.49	6.78	6.38	0.04	0.40	0.10	0.81	0.56	0.97
MCP^3^, mg/dL	61.48	56.48	58.61	60.90	1.05	0.10	0.89	0.02	<0.001	0.23
NH_3_-N, mg/dL	11.22^b^	11.58^ab^	13.01^a^	12.69^ab^	0.27	0.04	0.01	0.71	0.03	0.40
Total VFA, mmol/L	97.73	103.85	110.70	112.38	3.18	0.25	0.05	0.82	<0.001	0.10
Acetate, mmol/L	59.97	63.70	68.23	68.78	1.97	0.25	0.05	0.78	<0.001	0.08
Propionate, mmol/L	22.82	25.82	25.62	26.35	0.78	0.33	0.07	0.74	<0.001	0.11
Iso-butyrate, mmol/L	0.86	0.82	0.90	0.88	0.03	0.77	0.57	0.73	<0.001	0.48
Butyrate, mmol/L	11.11	11.47	12.51	12.76	0.40	0.33	0.07	0.93	0.002	0.31
Iso-valerate, mmol/L	1.60	1.55	1.79	1.91	0.07	0.11	0.03	0.35	<0.001	0.48
Valerate, mmol/L	1.38	1.49	1.64	1.69	0.05	0.05	0.007	0.91	<0.001	0.26
Acetate: propionate	2.69	2.65	2.72	2.60	0.04	0.72	0.55	0.65	0.59	0.67

a,b Means in the same row with different superscripts differ significantly (*p* < 0.05).

1 Treatments: CON = control group, basal diet; LCaP = low calcium propionate, basal diet plus 200 g/day calcium propionate; MCaP = medium calcium propionate, basal diet plus 350 g/day calcium propionate; and HCaP = high calcium propionate, basal diet plus 500 g/day calcium propionate.

2 SEM = Standard error of the mean.

3 MCP = Microbial crude protein.

### Sequencing Depth, Coverage, and Index of Bacterial Community

**Figure 1:**
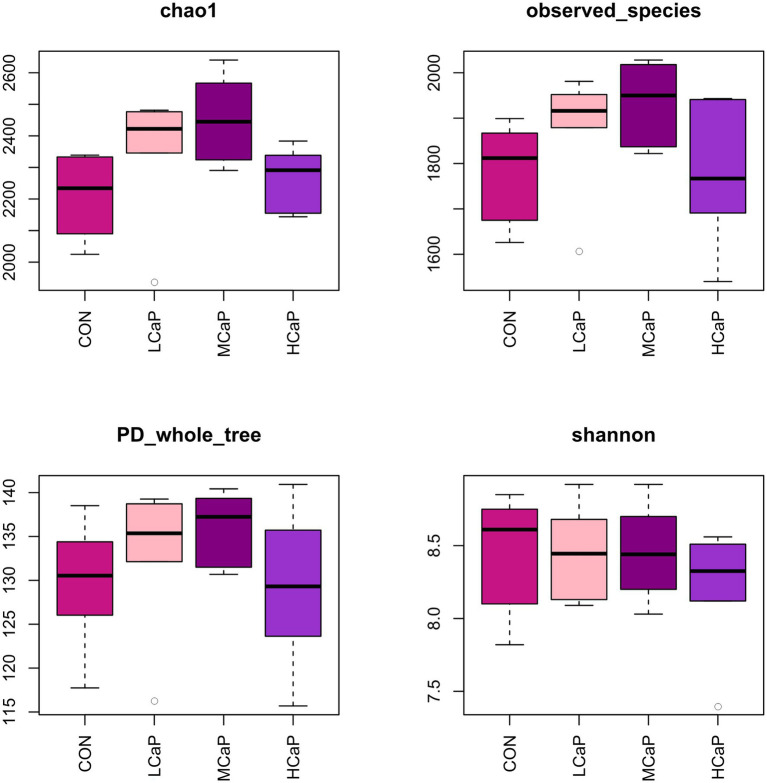
Alpha diversity of the rumen bacterial communities in dairy cows given different calcium propionate levels in early lactation. CON: control group, basal diet; LCaP: low calcium propionate, basal diet plus 200 g/day calcium propionate; MCaP: medium calcium propionate, basal diet plus 350 g/day calcium propionate; and HCaP: high calcium propionate, basal diet plus 500 g/day calcium propionate.

A total of 1,642,279 high quality V_3_-V_4_ 16S rRNA sequences were obtained from the 24 rumen samples, with an average of 68,428 sequence reads per sample (minimum 35,721; maximum, 163,397) after data quality checking, filtering, and removing of primers, chimeras, short sequences, and ambiguous bases. The length of the sequence reads was major distributed in 420–440 bp (Supplementary Table S2). In total, 3,442 OTUs were detected based on a similarity level ≥ 97%. The OUT numbers of CON, LCaP, MCaP, and HCaP groups were 2,938, 3,096, 3,025, and 2,928, respectively. The detailed common and unique OTUs numbers of ruminal bacteria among the four groups were shown in the Venn diagram (Supplementary Figure S1). The rarefaction curves (Supplementary Figure S2) of the rumen sample showed that the sequencing depth was sufficient and accurate to assess the diverse bacterial communities. The alpha diversity index analysis indicated that the supplementation of calcium propionate quadratically increased the Chao1 estimator (*p* = 0.02) and observed species (*p* = 0.03), but had no effect on the PD_whole_tree, Shannon index, and Simpson index (Figure 1; Supplementary Table S3).

The β-diversity was performed to compare the differences in microbial diversity among the treatments (Supplementary Figure S3). The PCoA and NMDS plots showed that the separation among the treatments of microbial diversity was not obvious at the OUT level.

### Rumen Bacterial Community Composition of Different Dietary Treatments

**Figure 2:**
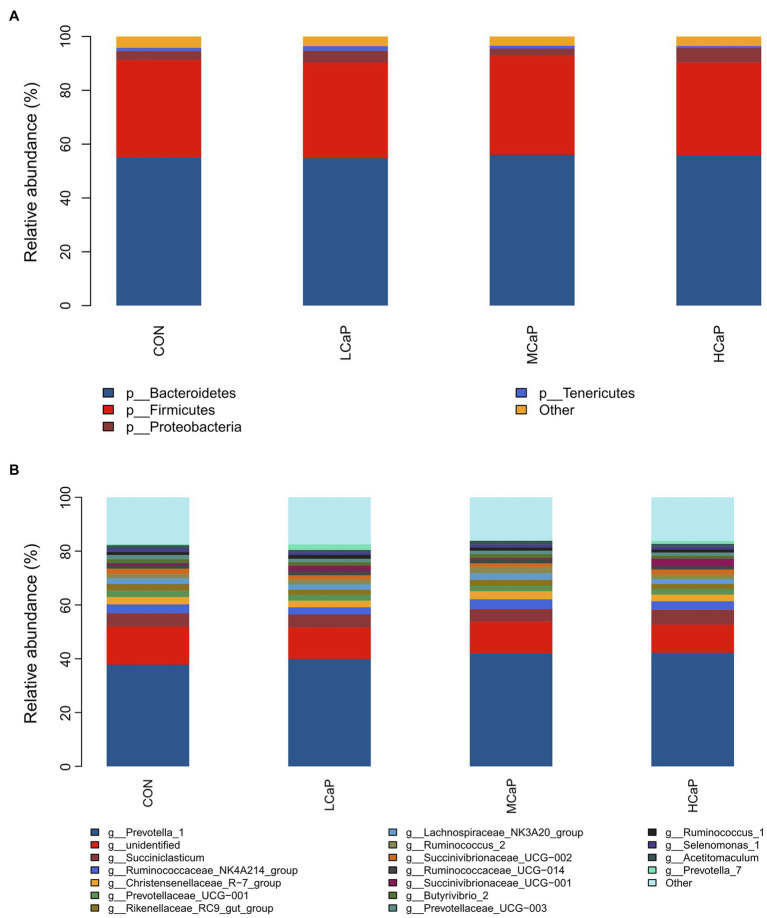
Distribution of the ruminal bacterial community composition across the four treatments. **(A)** Phylum level; **(B)** genus level. CON: control group, basal diet; LCaP: low calcium propionate, basal diet plus 200 g/day calcium propionate; MCaP: medium calcium propionate, basal diet plus 350 g/day calcium propionate; and HCaP: high calcium propionate, basal diet plus 500 g/day calcium propionate.

A comparison of the effects of calcium propionate supplementation on the rumen bacterial composition of early lactation dairy cows was performed by the taxonomic analysis. A total of 19 bacterial phyla were obtained from the 24 rumen samples. The most predominant phyla were *Bacteroidetes* (55.41% ± 1.56%, mean ± the SEM), *Firmicutes* (35.75% ± 1.34%), *Proteobacteria* (3.95% ± 0.58%), and *Tenericutes* (1.20% ± 0.17%) among the four groups (Figure 2A).

At the genus level, 254 genera belonging to 19 phyla were deleted in the samples. There were 16 most abundant shared genera with a relative abundance >1% in all the samples of the different treatments. Among these genera, *Prevotella_1* (40.46% ± 1.76%), *Succiniclasticum* (5.03% ± 0.42%), *Ruminococcaceae_NK4A214_group* (3.15% ± 0.21%), *Christensenellaceae_R-7_group* (2.65% ± 0.21%), *Prevotellaceae_UCG-001* (2.19% ± 0.16%), *Rikenellaceae_RC9_gut_group* (2.11% ± 0.16%), *Lachnospiraceae_NK3A20_group* (2.04% ± 0.16%), *Ruminococcus_2* (1.84% ± 0.19%), *Succinivibrionaceae_UCG-002* (1.74% ± 0.32%), *Ruminococcaceae_UCG-014* (1.50% ± 0.11%), *Succinivibrionaceae_UCG-001* (1.40% ± 0.48%), *Butyrivibrio_2* (1.40% ± 0.14%), *Prevotellaceae_UCG-003* (1.26% ± 0.08%), *Ruminococcus_1* (1.25% ± 0.10%), *Selenomonas_1* (1.10% ± 0.10%), and *Acetitomaculum* (1.10% ± 0.11%) were predominant across the four groups (Figure 2B).

As shown in Tables 3 , 4, different dietary calcium propionate supplement levels had little impact on the relative abundance of the major rumen bacteria compositions at phylum and genus level under the same basal dietary condition. The relative abundance of *Prevotellaceae_UCG-003* (*p* = 0.09) showed a trend linear decrease with the increasing calcium propionate supplementation to the early lactation dairy cows.

**Table 3:** Effects of dietary supplementation with calcium propionate on the relative abundance of ruminal bacterial communities at phylum level (average relative abundance ≥ 0.5% for at least one treatment) of dairy cows in early lactation.

		Treatments^1^			*p*-value^1^
Phylum					SEM^2^
	CON	LCaP	MCaP	HCaP		Treatment	Linear	Quadratic
*Bacteroidetes*	55.06	54.74	55.96	55.88	1.56	0.99	0.81	0.95
*Firmicutes*	36.15	35.57	36.82	34.46	1.34	0.95	0.77	0.77
*Proteobacteria*	3.40	5.34	2.71	4.35	0.58	0.43	0.44	0.52
*Tenericutes*	1.16	1.76	1.10	0.76	0.17	0.24	0.28	0.13
*Spirochaetae*	0.81	0.69	0.68	0.94	0.06	0.37	0.56	0.11
*SR1_Absconditabacteria*	0.80	0.62	0.56	0.67	0.09	0.84	0.59	0.48
*Actinobacteria*	0.73	0.74	0.64	0.56	0.06	0.72	0.31	0.67
*Saccharibacteria*	0.65	0.53	0.55	0.45	0.06	0.67	0.26	0.98
*Cyanobacteria*	0.59	0.58	0.48	0.52	0.06	0.93	0.61	0.90

1 Treatments: CON = control group, basal diet; LCaP = low calcium propionate, basal diet plus 200 g/day calcium propionate; MCaP = medium calcium propionate, basal diet plus 350 g/day calcium propionate; and HCaP = high calcium propionate, basal diet plus 500 g/day calcium propionate.

2 SEM = Standard error of the mean.

**Table 4:** Effects of dietary supplementation with calcium propionate on the relative abundance of ruminal bacterial communities at genera level (average relative abundance ≥ 0.5% for at least one treatment) of dairy cows in early lactation.

		Treatments^1^			*p*-value	
Phylum	Genus					SEM^2^	
		CON	LCaP	MCaP	HCaP		Treatment	Linear	Quadratic
*Bacteroidetes*	*Prevotella_1*	37.77	39.88	42.00	42.20	1.76	0.81	0.36	0.86
	*Prevotellaceae_UCG-001*	2.37	2.25	2.02	2.11	0.16	0.88	0.49	0.80
	*Prevotellaceae_UCG-003*	1.51	1.21	1.22	1.10	0.08	0.32	0.09	0.61
	*Pseudobutyrivibrio*	0.69	0.77	0.77	0.73	0.06	0.98	0.81	0.71
	*Rikenellaceae_RC9_gut_group*	2.48	1.81	2.25	1.90	0.16	0.42	0.33	0.59
*Firmicutes*	*Acetitomaculum*	1.23	0.83	1.39	0.95	0.11	0.29	0.73	0.94
	*Butyrivibrio_2*	1.49	1.37	1.57	1.15	0.14	0.77	0.55	0.63
	*Christensenellaceae_R-7_group*	2.71	2.46	2.99	2.45	0.21	0.79	0.89	0.79
	*Eubacterium_coprostanoligenes_grou*	0.62	0.48	0.55	0.46	0.03	0.24	0.11	0.68
	*Eubacterium_ruminantium_group*	0.67	0.63	0.61	0.57	0.05	0.92	0.50	0.94
	*Lachnospiraceae_NK3A20_group*	2.07	2.01	2.34	1.75	0.16	0.67	0.71	0.47
	*Ruminococcaceae_NK4A214_group*	3.26	2.66	3.59	3.11	0.21	0.50	0.85	0.77
	*Ruminococcaceae_UCG-005*	0.65	0.66	0.74	0.50	0.06	0.59	0.54	0.32
	*Ruminococcaceae_UCG-014*	1.55	1.71	1.42	1.34	0.11	0.68	0.40	0.52
	*Ruminococcus_1*	1.15	1.44	1.24	1.17	0.10	0.77	0.97	0.38
	*Ruminococcus_2*	1.61	1.74	2.35	1.67	0.19	0.51	0.63	0.36
	*Saccharofermentans*	0.75	0.68	0.72	0.58	0.05	0.71	0.37	0.73
	*Schwartzia*	0.59	0.47	0.39	0.60	0.07	0.70	0.91	0.29
	*Selenomonas_1*	1.28	0.98	1.05	1.09	0.10	0.76	0.55	0.41
	*Succiniclasticum*	4.94	4.83	4.71	5.64	0.42	0.88	0.64	0.56
*Proteobacteria*	*Succinivibrionaceae_UCG-001*	0.52	1.87	0.49	2.72	0.48	0.29	0.22	0.68
	*Succinivibrionaceae_UCG-002*	2.06	1.61	1.37	1.90	0.32	0.89	0.78	0.50
*Saccharibacteria*	*Candidatus_Saccharimonas*	0.65	0.53	0.55	0.45	0.06	0.67	0.26	0.98
*Spirochaetae*	*Treponema_2*	0.80	0.67	0.67	0.93	0.06	0.36	0.54	0.10

1 Treatments: CON = control group, basal diet; LCaP = low calcium propionate, basal diet plus 200 g/day calcium propionate; MCaP = medium calcium propionate, basal diet plus 350 g/day calcium propionate; and HCaP = high calcium propionate, basal diet plus 500 g/day calcium propionate.

2 SEM = Standard error of the mean.

## Discussion

Calcium propionate is widely used as a food and feed additive to inhibit mold or as a nutrition additive. Ruminal fermentation and rumen bacterial community play key roles in the production performance and health of dairy cows. The purpose of the study was to evaluate the effects of different dietary calcium propionate feeding levels, with the same TMR, on ruminal fermentation parameters and bacterial community composition in Holstein dairy cows by 16S rRNA high-throughput sequencing technology.

The fermentation characteristics of ruminal pH, MCP, NH_3_-N, and VFA concentration can reflect the function and the stability of the internal environment in the rumen. The ruminal pH is an important indicator of proper rumen fermentation and health (Zhang et al., 2018). Lee-Rangel et al. (2012) and Zhang et al. (2018) reported similar results that rumen pH was not affected by calcium propionate supplementation. The increased total VFA concentration is commonly associated with lower rumen pH (Fregulia et al., 2021). But in this study, the increasing total VFA concentration did not change the rumen pH may be related to the weakly alkaline properties of calcium propionate aqueous solution (Zhang et al., 2020). The unchanged rumen pH indicated that the calcium propionate supplementation did not affect the rumen normal function.

Ruminal NH_3_-N concentration is the balance between dietary protein degradation, and microbial utilization (Jiao et al., 2021). Liu et al. (2009) found that the ruminal CP degradability of concentration mix was linearly and quadratically increased with greater calcium propionate supplementation in finishing steers. The increased CP degradation resulted in a higher ruminal NH_3_-N concentration (Unnawong et al., 2021). The linearly increased concentration of ruminal NH_3_-N and the quadratically decreased MCP concentration in the calcium propionate treatment groups indicated that less ruminal NH_3_-N was incorporated into MCP or the MCP consumption increased in the treatments. Propionate is a great contributor to gluconeogenesis and supports milk synthesis for dairy cows (Reynolds et al., 2003). Miettinen and Huhtanen (1996) and Sheperd and Combs (1998) observed propionate infusion improved the milk and protein yield. The dairy cows fed calcium propionate had more milk yield and milk protein levels (Martins et al., 2019; Zhang et al., 2022), which improved the protein requirement during early lactation. Therefore, it was speculated that improving milk and protein yield might result in increasing MCP consumption by improving MCP flow to small intestines.

The VFAs, also known as short-chain fatty acids, are produced by microbial fermentation of carbohydrates and endogenous substrates and supply approximately 70% of the energy required to ruminants (Bergman, 1990). The increased ruminal VFAs concentration in the calcium propionate treatment groups stimulated the capacity for VFA absorption (Dieho et al., 2017), which was beneficial to alleviate NEB for early lactation dairy cows. It was reported that calcium propionate supplementation in calves could increase the rumen acetate, propionate, and butyrate concentrations (Zhang et al., 2018). Similarly, in the present study, it was found that the concentrations of acetate, propionate, butyrate, iso-valerate, and valerate were increasing in the calcium propionate supplementation groups. As a precursor of glucose, the advantage of propionate feeding to dairy cows is that it can improve milk production and feed intake (McNamara and Valdez, 2005). Cifuentes-Lopez et al. (2021) also observed the rumen total VFA increased linearly as dietary calcium propionate increased in fishing lambs. Liu et al. (2009) indicated that the calcium propionate supplementation improved the rumen fermentation and the *in situ* ruminal degradation of organic matter, NDF, and CP in beef cattle. Our previous study also showed that the dry matter intake of dairy cows showed a quadratic increase with the increasing supplementation of calcium propionate (Zhang et al., 2022). It was accepted that the increased feed intake and rumen nutrient digestibility improved the ruminal VFAs concentration of dairy cows in early lactation.

Calcium propionate is dissociated to Ca^2+^ and propionic acid in rumen aqueous solutions (Zhang et al., 2018). In this study, the exogenous supplementation of calcium propionate did not improve the molar proportion of propionate. Propionate acted as a gluconeogenic precursor (Kennedy et al., 2020) and a signaling molecule to stimulate rumen development (Sakata and Tamate, 1979; Zhang et al., 2018). The growth and integrity of the rumen epithelium were important for the absorption of VFAs (Zebeli et al., 2015). Therefore, it was speculated that the unchanged molar proportion of propionate may be related to the sampling time and the improvement of rumen epithelium absorption capacity. The rapid ruminal propionate absorption resulted in a brief increase in propionate molar proportion after feeding, but not changed at the sampling time (before morning feeding). With the increasing absorption of propionate as glucose precursors, we had found that feeding calcium propionate improves milk production of dairy cows in the previous study (Zhang et al., 2022). Lee-Rangel et al. (2012) also indicated the proportion of rumen acetate, propionate, and butyrate were not affected by the addition of calcium propionate in the diet of lambs. The increased total concentration of VFA and the unchanged molar proportion of acetate, propionate, and butyrate indicated that calcium propionate supplementation can improve rumen VFA production, but had minimal effects on fermentation mode.

There was little reported previously about the effects of calcium propionate feeding levels on the ruminal bacterial community of dairy cows in early lactation by pyrosequencing of the 16S RNA gene. The alpha diversity of bacterial communities was evaluated by quantitative methods. It showed that the calcium propionate feeding quadratically increased the Chao 1 index and the observed_species, which suggested that the 350 g/day calcium propionate feeding level may improve bacterial diversity and richness in the rumen. However, compared with the MCaP group, the Chao 1 index and the observed_species in the HCaP group were decreasing, indicating the feeding level of 500 g/day exceeded the optimal feeding level. The relative abundance of rumen bacterial genera has a significant correlation with the apparent digestibility of nutrients and rumen fermentation characteristics (He et al., 2018). Feeding calcium propionate improved the alpha diversity of the rumen bacterial community, which explained the increases in rumen NH_3_-N and the total VFA concentration of the calcium propionate treatment groups.

The PCoA and NMDS results also verified no significant differences in ruminal bacterial community structures among the four groups. In this study, the relative abundance of dominant phyla and the majority of the genera with the relative abundance ≥ 0.5% were not affected by the calcium propionate treatments. He et al. (2018) found that calcium salt of long-chain fatty acids increased microbial diversity index, but no significant difference in bacteria abundance at the genus level was found in Holstein bulls. The data of Yao et al. (2017) suggested that the ruminal bacterial community composition was nearly unchanged by calcium propionate supplementation in finishing bulls. The dietary changes have important impacts on rumen bacterial communities, but the dietary forage to concentrate ratio was the main factor affecting the rumen microbial population structure under the same diet ingredients and processing conditions (Bi et al., 2018). It was reported the rapid transition to a high-grain diet resulted in dynamic changes in the sheep rumen microbiome (Seddik et al., 2019). Bi et al. (2018) reported that an 8% difference in dietary energy levels had little impact on rumen bacterial community composition in heifers. In contrast, Cao et al. (2020) indicated calcium propionate supplementation decreased the diversity of bacteria and altered the ruminal microbiota in calves both pre- and postweaning. The difference may be related to the experimental animals and basal diet. Propionic acid and calcium ions were normally present in rumen. The early lactation dairy cows required large amounts of propionic acid and calcium ions for alleviating NEB and hypocalcemia, respectively. After calcium propionate was rapidly absorbed by the rumen of dairy cows for milk synthesis, the composition of rumen fluid remained relatively balanced. Therefore, the relative abundance of the majority rumen microbiota composition was not affected by the different calcium propionate feeding levels. This study showed no difference was found in the relative abundance of ruminal bacterial community composition among the different calcium propionate feeding levels, which was also consistent with the similar molar proportions of VFA among the treatments.

The *Bacteroidetes*, *Firmicutes*, and *Proteobacteria* were the dominant phyla in the rumen of the study, which was similar to the previous study (Wang et al., 2019, 2021c). The sequence analysis of the reads from the four treatments showed the phylum *Bacteroidetes* was the dominant bacteria, which was mainly composed of the genus *Prevotella_1*. Many reports suggested that the *Prevotella* genus was the dominant ruminal genus in the abundance of dairy cows (He et al., 2018; Mu et al., 2021), which was similar to the results of ours. *Prevotella*, which can degrade starch and protein, had many OTUs strongly associated with feed efficiency (Fregulia et al., 2021). In our study, the relative abundance of *Prevotella_1* increased with the supplementation of calcium propionate, but there were no significant differences among the four groups. The NH_3_-N concentration was significantly positively correlated with the relative abundance of *Prevotella_1* (Bi et al., 2018; Cao et al., 2020), and the numerical increase in relative abundance was likely linked to the higher degradation of protein and greater NH_3_-N production (He et al., 2018). *Succiniclasticum* was another prevalent genus in this study. And the high relative abundance of *Succiniclasticum* was commonly observed in dairy cows fed high levels of concentration (Bi et al., 2018; Wang et al., 2021a). The results of the present study were similar to the report of Yao et al. (2017), in which, the relative abundance of *Succiniclasticum* was also not affected by calcium propionate supplementation. The dominant rumen microorganisms were not significantly different among the four groups eventually led to no changed molar proportions of VFA.

In general, there was no significant change in the abundance of microbial community composition at phyla and genus levels, which may be due to the same dietary TMR, indicating that dietary calcium propionate feeding level did not affect the rumen bacterial community.

## Conclusion

This study demonstrated that the supplementation of calcium propionate to the dairy cows in early lactation had no significant impact on major rumen bacterial community composition under the same basal TMR diet. Furthermore, *Bacteroidetes*, *Firmicutes*, and *Proteobacteria* were the dominant phyla in all the treatments. However, the calcium propionate supplementation increased the alpha diversity of the rumen bacterial communities and improved rumen fermentation of the dairy cows. This study is useful to guide future research on investigating the calcium propionate application in alleviating NEB and hypocalcemia of early lactation dairy cows.

## Data Availability Statement

The datasets presented in this study can be found in online repositories. The names of the repository/repositories and accession number(s) can be found in the article/Supplementary Material.

## Ethics Statement

The animal study was reviewed and approved by Animal Care Committee of the Chinese Academy of Agricultural Sciences (Beijing, China).

## Author Contributions

FZ, YG, and BX conceived and designed the experiment. FZ and YW were involved in the animal experiment and sample collection. FZ analyzed the data and completed the initial manuscript. HW and XN completed the language editing and revised the manuscript. BX provided funding support. All authors contributed to the article and approved the submitted version.

## Funding

This study was financially supported by the Central Public-Interest Scientific Institution Basal Research Fund (No. 2019-YWF-YTS-10), State Key Laboratory of Animal Nutrition (2004DA125184G2104), and Beijing Dairy Industry Innovation Team (bjcystx-ny-1).

## Conflict of Interest

The authors declare that the research was conducted in the absence of any commercial or financial relationships that could be construed as a potential conflict of interest.

## Publisher’s Note

All claims expressed in this article are solely those of the authors and do not necessarily represent those of their affiliated organizations, or those of the publisher, the editors and the reviewers. Any product that may be evaluated in this article, or claim that may be made by its manufacturer, is not guaranteed or endorsed by the publisher.

## Footnotes

^1^ https://www.ncbi.nlm.nih.gov/sra/PRJNA791506
