# Supplementary material for: Calcium Propionate Supplementation Has Minor Effects on Major Ruminal Bacterial Community Composition of Early Lactation Dairy Cows
Source: Front Microbiol. 2022 Mar 8;13:847488. doi: 10.3389/fmicb.2022.847488 (PMC8964349; doi:10.3389/fmicb.2022.847488)
Supplement: Supplementary file 1 [file Data_Sheet_1.docx]

**Calcium propionate supplementation has minor effects on major ruminal bacterial community composition of early lactation dairy cows**

**Fan Zhang^1,2^, Yue Wang^1^, Hui wang^1^, Xuemei Nan^1^, Yuming Guo^2,*^ and Benhai Xiong^1,^***

^1^ State Key Laboratory of Animal Nutrition, Institute of Animal Science, Chinese Academy of Agricultural Sciences, Beijing 100193, China

^2^ State Key Laboratory of Animal Nutrition, College of Animal Science and Technology, China Agricultural University, Beijing 100193, China

***** Corresponding author: Yuming Guo, Email: guoyum@cau.edu.cn; Benhai Xiong, Email: xiongbenhai@caas.cn

**Supplementary Table S1**. Effect of increasing supplementation of calcium propionate in the molar proportions of individual rumen fluid VFA of dairy cows in early lactation

| Items | Treatments ^1^ | | | | SEM ^2^ | *P*-value | | |  |  |
| --- | --- | --- | --- | --- | --- | --- | --- | --- | --- | --- |
|  | CON | LCaP | MCaP | HCaP |  | Treatment | Linear | Quadratic | Week | Treatment × Week |
| Acetate, (mol/100 mol) | 61.55 | 61.61 | 61.83 | 60.84 | 0.23 | 0.48 | 0.40 | 0.26 | 0.45 | 0.46 |
| Propionate, (mol/100 mol) | 23.29 | 23.68 | 23.00 | 23.82 | 0.25 | 0.66 | 0.68 | 0.74 | 0.64 | 0.64 |
| Iso-butyrate, (mol/100 mol) | 0.88 | 0.80 | 0.81 | 0.79 | 0.02 | 0.79 | 0.07 | 0.31 | 0.01 | 0.19 |
| Butyrate, (mol/100 mol) | 11.24 | 10.98 | 11.27 | 11.36 | 0.10 | 0.63 | 0.56 | 0.35 | 0.67 | 0.68 |
| Iso-valerate, (mol/100 mol) | 1.64 | 1.49 | 1.60 | 1.69 | 0.03 | 0.18 | 0.46 | 0.06 | 0.04 | 0.99 |
| Valerate, (mol/100 mol) | 1.41 | 1.44 | 1.48 | 1.51 | 0.01 | 0.04 | 0.004 | 0.90 | 0.16 | 0.39 |

^1^Treatments: CON = control group, basal diet; LCaP = low calcium propionate, basal diet plus 200 g/d calcium propionate; MCaP = medium calcium propionate, basal diet plus 350 g/d calcium propionate; HCaP = high calcium propionate, basal diet plus 500 g/d calcium propionate.

^2^SEM = Standard error of the mean.

**Supplementary Table S2**. Distribution statistics of high-quality sequences

| Distribute | Reads (bp) |
| --- | --- |
| 0-200 | 0 |
| 200-260 | 33 |
| 260-320 | 818 |
| 320-360 | 15 |
| 360-380 | 7 |
| 380-400 | 693 |
| 400-420 | 501,820 |
| 420-440 | 1,138,889 |
| 440-460 | 4 |
| 460-480 | 0 |
| 480-500 | 0 |
| 520-540 | 0 |
| 540-560 | 0 |
| 560-600 | 0 |

**Supplementary Table S3**. Alpha diversity of the rumen bacterial communities in dairy cows given different calcium propionate levels in early lactation.

| Items | Treatmentsn ^1^ | | | | SEM ^2^ | *P*-value | | |
| --- | --- | --- | --- | --- | --- | --- | --- | --- |
|  | CON | LCaP | MCaP | HCaP |  | Treatment | Linear | Quadratic |
| Chao 1 | 2,209 ^b^ | 2,348 ^ab^ | 2,452 ^a^ | 2,267 ^ab^ | 33.91 | 0.05 | 0.25 | 0.02 |
| Observed_species | 1,782 | 1,875 | 1,934 | 1,775 | 27.90 | 0.12 | 0.76 | 0.03 |
| PD_whole_tree | 129.6 | 132.9 | 136.1 | 129.1 | 1.56 | 0.38 | 0.84 | 0.13 |
| Shannon | 8.46 | 8.45 | 8.46 | 8.21 | 0.07 | 0.58 | 0.30 | 0.41 |
| Simpson | 0.99 | 0.99 | 0.99 | 0.99 | 0.001 | 0.54 | 0.61 | 0.19 |

^a,b^Means in the same row with different superscripts differ significantly (*P* < 0.05).

^1^Treatments: CON = control group, basal diet; LCaP = low calcium propionate, basal diet plus 200 g/d calcium propionate; MCaP = medium calcium propionate, basal diet plus 350 g/d calcium propionate; HCaP = high calcium propionate, basal diet plus 500 g/d calcium propionate.

^2^SEM = Standard error of the mean.


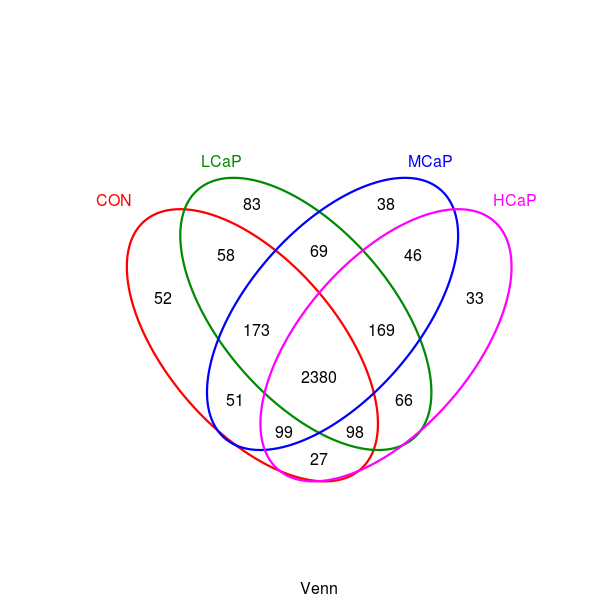


**Supplementary Figure S1**. Common and unique OTU number of ruminal bacteria in CON, LCaP, MCaP, and HCaP group. CON: control group, basal diet; LCaP: low calcium propionate, basal diet plus 200 g/d calcium propionate; MCaP: medium calcium propionate, basal diet plus 350 g/d calcium propionate; HCaP: high calcium propionate, basal diet plus 500 g/d calcium propionate.


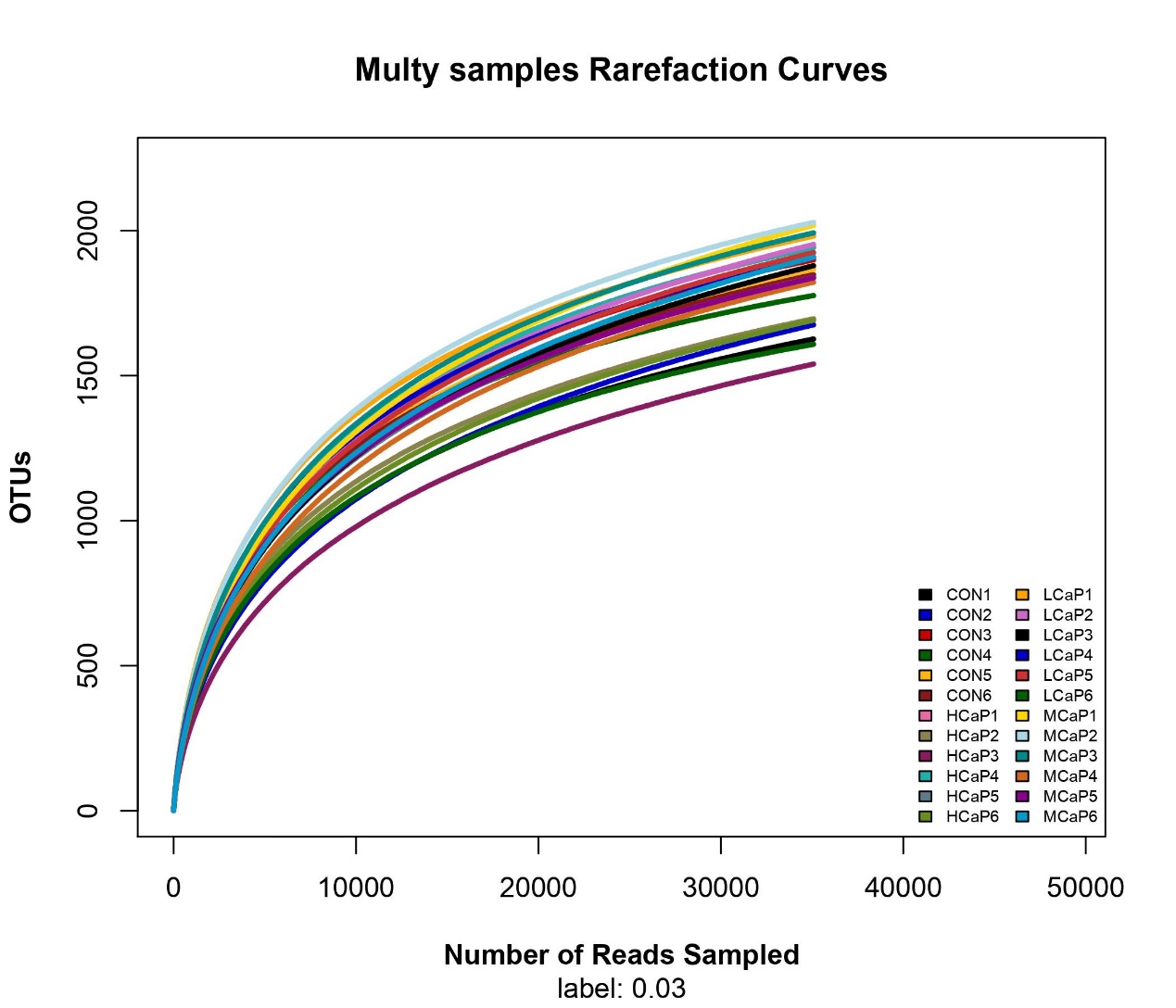


**Supplementary Figure S2**. Rarefaction curves of OTU number in rumen fluid samples. CON: control group, basal diet; LCaP: low calcium propionate, basal diet plus 200 g/d calcium propionate; MCaP: medium calcium propionate, basal diet plus 350 g/d calcium propionate; HCaP: high calcium propionate, basal diet plus 500 g/d calcium propionate.

A


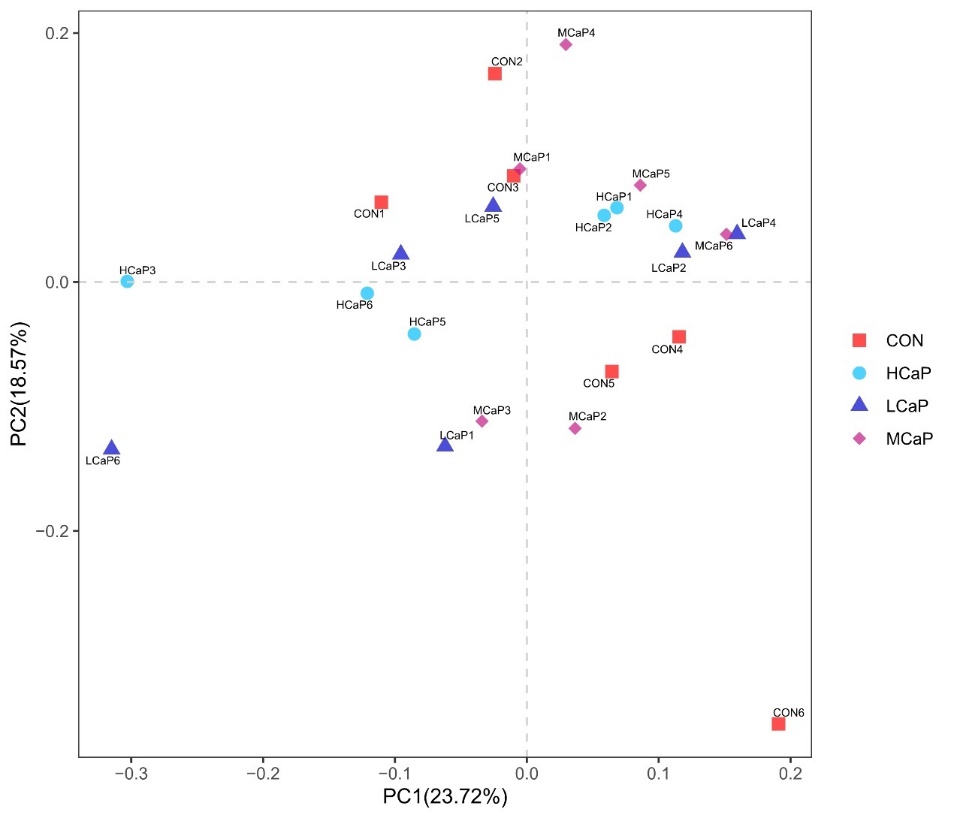


B


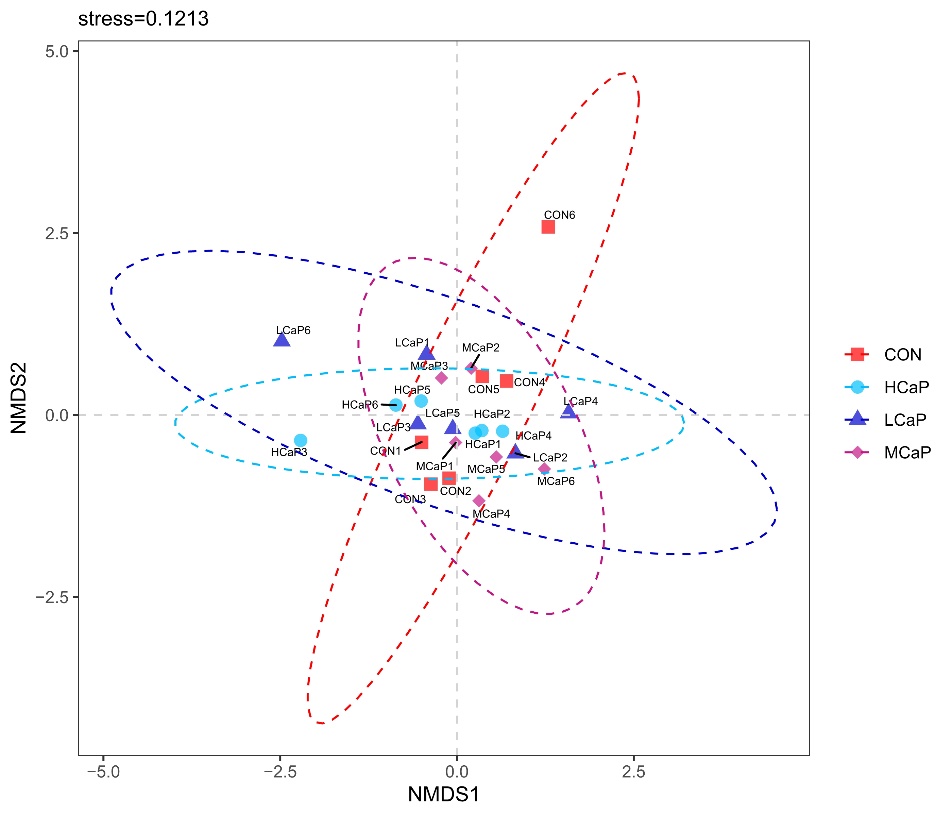


**Supplementary Figure S3** Beta community diversity analysis of ruminal microbiota of (A) principal coordinate analysis (PCoA) and (B) non-metric multidimensional scaling analysis (NMDS) of the four treatments. CON: control group, basal diet; LCaP: low calcium propionate, basal diet plus 200 g/d calcium propionate; MCaP: medium calcium propionate, basal diet plus 350 g/d calcium propionate; HCaP: high calcium propionate, basal diet plus 500 g/d calcium propionate.
